# Ectomycorrhizal morphology and soil nutrient status control the C: N: P: K stoichiometry in Faxon fir (*Abies fargesii* var. *faxoniana*), in the subalpine forests of Southwest China

**DOI:** 10.3389/fpls.2025.1549476

**Published:** 2025-05-16

**Authors:** Lulu Chen, Xuhua Li, Zuoxin Tang

**Affiliations:** ^1^ Institute of Geographic Sciences and Natural Resources Research, Chinese Academy of Sciences (CAS), Beijing, China; ^2^ Sichuan Provincial Key Laboratory of Ecological Restoration and Conservation for Forest and Wetland, Sichuan Academy of Forestry Sciences, Chengdu, China; ^3^ College of Agricultural and Life Sciences, Kunming University, Kunming, Yunnan, China

**Keywords:** stoichiometric ratios, nutrient allocation, ectomycorrhizal traits, ectomycorrhizal morphology, subalpine forest, *Abies fargesii* var. *faxoniana*

## Abstract

The stoichiometry of plant carbon (C), nitrogen (N), phosphorus (P), and potassium (K) provides insights into the nutritional and growth strategies of trees in changing environments. However, abiotic and biotic effects on nutrient stoichiometry in trees of subalpine forests, in the context of climate change, are not fully understood. We focused on the dominant tree species, Faxon fir (*Abies fargesii* var. *faxoniana*) located on the eastern edge of the Tibetan Plateau to examine the dynamics and balance of C, N, P, and K in plant tissues, and their driving factors across different habitats. As this tree is typically associated with ectomycorrhizal fungi, we compiled a new dataset of ectomycorrhizal functional traits to evaluate their importance in tree-nutrient stoichiometry. We found that K was the most limiting nutrient in the roots (1.1 ± 0.08 mg g^-1^) and foliage (10.8 ± 0.3 mg g^-1^) in Faxon fir and probably the main factor for productivity constraints. Faxon fir exhibited preferential allocation of N, P, and K nutrients to leaves in contrast to roots. Variations in C:N:P:K stoichiometry were primarily explained by soil variables, followed by ectomycorrhizal traits. Specifically, foliar nutrient stoichiometry was closely associated with the formation and morphological traits of ectomycorrhizal root tips, whereas root nutrient stoichiometry was related to the one- (length) and two- (area) dimensional foraging spaces radiated by the ectomycorrhizal mycelium. Our findings demonstrate an imbalance in C:N:P:K stoichiometry in Faxon fir within the changing environments of subalpine ecosystems and highlight the crucial regulatory role of ectomycorrhizal functional traits in mediating these imbalances.

## Introduction

Plant carbon (C), nitrogen (N), phosphorus (P), and potassium (K) stoichiometry reflects ecological adaptations to environmental gradients, yet how these traits vary in individual tree species—particularly in climate-sensitive high-elevation forests—remains poorly understood (Reich and Oleksyn, 2004; Sardans et al., 2011; Tian et al., 2019).While C-N-P stoichiometry is widely studied (Elser et al., 2007; Hessen et al., 2004), K—critical for photosynthesis and osmotic regulation (Leigh and Wyn Jones, 1984; Sardans et al., 2012)—is often overlooked, despite its role in metabolic balance and plant production (Sardans and Peñuelas, 2015). C:N:P:K ratios reflect nutrient availability and limitations of trees (Elser et al., 2007; Güsewell, 2004). For example, low N:P ratios (<14) suggest N limitation, while high N:P (>16) indicates P limitation (Koerselman and Meuleman, 1996). Integrated C:N:P:K ratios could better elucidate nutrient limitations and tree growth strategies under global change (Elser et al., 2010; Finzi et al., 2011). Considering the high climate sensitivity of forests at high elevations (Beniston et al., 1997; Körner, 1998), the balance of plant C, N, P, and K nutrients and their stoichiometry is important for maintaining the production and stability of forest ecosystems under global change (Fernández-Martínez et al., 2014). To date, few studies have dealt with the patterns and driving factors of C, N, P, and K stoichiometry in high-elevation forest trees confronting nutrient availability limitations.

Concurrently, the C, N, P, and K allocation strategies in different organs reflect the resource utilization strategies and adaptation mechanisms of trees in changing environments (Kleyer and Minden, 2015; Reich et al., 2008; Zhang et al., 2020). Indeed, the productivity of tree species is largely regulated by nutrient allocation to the various sink tissues in different organs (Poorter et al., 2012; Sardans and Peñuelas, 2013; Tang et al., 2018), such that, to sustain a functional balance and overall metabolic stability in a changing environment, trees selectively regulate the nutrient composition of sink tissues and organs by avoiding excessive fluctuations in the contents of important and scarce elements (Schreeg et al., 2014; Yan et al., 2016). Thus, the stability of element stoichiometry in their different organs reflects the growth dynamics of trees in changing environments (Hu et al., 2021).

Numerous environmental constraints in mountain ecosystems, such as soil nutrient scarcity (Zhang et al., 2020), low temperature (Reich and Oleksyn, 2004; Yuan and Chen, 2009), and moisture limitations (Sardans et al., 2008a, b), collectively reducing nutrient acquisition efficiency and slowing growth of trees (Körner, 2003; Reich and Oleksyn, 2004). These factors variably influence tree nutritional status across elevational gradients (Wang et al., 2020; Wright et al., 2004; Zhang et al., 2020). Plant tissue nutrient-element stoichiometry could be determined by soil fertility (Müller et al., 2017; Sardans et al., 2012; Vitousek et al., 2010); furthermore, the relationships of nutrient element stoichiometry between plants and soil can allegedly predict the instability status of plants (Elser et al., 2010; Tian et al., 2024; Zheng et al., 2021). Several hypotheses, including temperature-biogeochemistry- and temperature-plant physiology-based hypotheses, have been proposed to account for the influence of climate factors on various metabolic processes and nutrient acquisition efficiency of trees (McGroddy et al., 2004; Reich and Oleksyn, 2004). The stoichiometric homeostasis and element stability of trees can be altered by environmental factors (Hu et al., 2021; Wan et al., 2023), reflecting tree adaptation strategies. Thus, predicting plant C-N-P-K stoichiometry during tree growth under climate change is vital, particularly in sensitive mountain ecosystems.

Biotic factors like root nutrient uptake (Elser et al., 2007; Lambers, 2022; Lugli et al., 2020) greatly influence tree chemical traits (Sardans and Peñuelas, 2013; Tang et al., 2018). Notably, ectomycorrhizal fungi (ECM) associated with roots facilitate N and P uptake in conifers in exchange for plant carbon (Smith and Read, 2010). This C-nutrient trade between conifer tree species and ECM fungi involves C-nutrient metabolism and homeostasis in the host (Bunn et al., 2024). Numerous studies have shown that plant N and P stoichiometry is closely correlated with ECM functional traits (Chen et al., 2021; Michelsen et al., 1996; Nehls and Plassard, 2018), such as the frequency of occurrence of ECM fungi. Specifically, K^+^ acquisition by trees can be regulated by the hydrophilicity or hydrophobicity of the ECM mycelium (van der Linde et al., 2018). Indeed, K has been shown to play a role in substance transfer between roots and ECM fungi (Wallander and Wickman, 1999), but the issue has scarcely been explored. ECM fungi that differentiate abundant extraradical mycelia play an important role in host nutrient acquisition (Agerer, 2001; Weigt et al., 2012) and are closely linked to host nutrition conditions (Chen et al., 2021; Ostonen et al., 2011, 2017). However, to date, few studies have focused on the relationships between ECM functional traits and C, N, P, and K nutrients in tree species, and only a few useful ECM indicators are known that are effective predictors of tree nutrient stoichiometry.

Here, we focused on the *Abies* tree species, Faxon fir (*Abies fargesii* var. *faxoniana*), which dominates subalpine forests on the eastern edge of the Tibetan Plateau. The habitat of this tree species encompasses the primary distribution area of giant pandas; therefore, the growth and population development of Faxon fir are crucial for the overall stability of regional ecosystems (Pauli et al., 2012; Taylor et al., 2006). Understanding its nutrient dynamics is therefore essential for ecosystem conservation and stability. However, the dynamics of the C, N, P, and K nutrients and the corresponding driving mechanisms in this species along environmental gradients have been poorly explored. Given the importance of ECM fungi for tree adaptation to high-elevation habitats (Argüelles-Moyao and Garibay-Orijel, 2018; Chen et al., 2021), we investigated the patterns of C, N, P, and K element stoichiometry in Faxon fir along an environmental gradient and explored the importance of ECM associations with these nutrient elements in addition to abiotic (climate and soil conditions) factors. Importantly, we attempted to build a new dataset of ECM functional traits to test ECM predictability for nutrient stoichiometry of Faxon fir. We aimed to answer the following questions: 1) How do nutrient elements in Faxon fir plant tissue (roots and leaves) vary along environmental gradients? 2) How and to what extent do biotic (ECM traits) and abiotic (climatic and soil conditions) factors affect the patterns and dynamics of plant C:N:P:K stoichiometry? 3) Are ECM functional traits predictive of C:N:P:K stoichiometry in Faxon fir?

## Materials and methods

### Filed survey and study sites

Sampling and field experiments were conducted in the Wolong Nature Reserve (30°51’N, 102°58’12’’E, WoL), the Miyaluo Nature Reserve (31° 42’N, 102° 46’E, MiyL), and the Wanglang Nature Reserve (33°00′N, 104°01′E, WaL), in Sichuan Province, China, where Faxon fir is continuously distributed in clusters (Chen et al., 2021). Five sites at the following elevations: 2850, 3000, 3194, 3413, and 3593 m asl., were selected in WoL, and two more, of clustering and upper bound distributions in MiyL (3077 and 3600 m asl.), and WaL (3070, 3150 m asl.). The latter two sites are high-mountain regions with deep valleys, where Faxon fir populations predominantly occur in areas over 3000 m asl. All these sites are located on the eastern flanks of the Qinghai-Tibetan Plateau and are characterized by dry, cold winters, and wet, cool summers (Li et al., 2020, 2013; Zhao et al., 2012). Faxon fir is the dominant tree species in the three study areas in coexistence with other species, such as *Rhododendron* spp., *Picea purpurea* Mast, *Betula albosinensis* Burkill, and *Betula platyphylla* Suk.

The climate dataset for our study sites was extracted from the national grid data derived from observations covering more than 2400 meteorological stations and constructed by the “anomaly approach” (New et al., 2000; Xu et al., 2009). Finally, we obtained mean annual temperature (MAT) and mean annual precipitation (MAP) datasets for each study site. MAT for each site was calculated with an air temperature-lapse rate of 0.65°C 100 m^-1^ (Chen et al., 2021; Zhao et al., 2008).

### Field sampling and processing

Leaf, root, and soil samples were collected from nine elevations at the three study sites from June to August 2018. Using the point-centered quarter-sampling method, eight equidistant focal trees per elevation were randomly selected as center points with a horizontal transect of *c*.100 m (Mitchell, 2010). Four trees with a diameter at breast height of 35–60 cm were sampled at each center point from different quarters; these trees were all within 10 m of the focal tree, resulting in 32 sub-samples collected at each elevational site, for a total of 288 sub-samples for the nine experimental elevations. A cubic soil block of 10 cm × 10 cm × 10 cm near the lateral roots, 1 m away from each target tree, was collected at each site, as described by Chen et al. (2021). Fine roots with a diameter < 2 mm were selected from each soil block for subsequent processing. Fine roots in Faxon fir trees are easily distinguishable by their reddish-brown color and particular aromatic scent.

In the laboratory, two random root samples for each center point were gently cleaned by washing with deionized water and stored in 5% glycerin at −20°C for ECM identification following the method of Köhler et al. (2018). The other two samples were oven-dried at 48°C to constant weight and used for measurement of root dry biomass and chemical trait analysis. Fresh soil samples were frozen-stored at −20°C until further processing and analysis. Leaves from four trees belonging to one central point were mixed for analysis of C, N, P, and K.

### Plant and soil chemistry analysis

Root and leaf samples were cleaned with deionized water, dried to a constant weight at 60°C, and crushed through a 60-mesh sieve to prepare for analytical determination of C, N, P, and K concentrations. After grinding and milling (ball mixer-mill MM400, Retsch, Germany), oven-dried fine root and leaf samples (approximately 10 mg) were prepared for the analysis of root C and N concentrations using an elemental analyzer (Vario EL III, CHNOS Elemental Analyzer; Elementar Analysensysteme GmbH, Germany). In turn, K and P concentrations in were measured using an ICP (ICAP6300). Then, tree C:N, C:P, N:P, N:K, and P:K ratios were calculated (**Table 1**).

**Table 1:** Descriptive statistics of root and foliar C, N, P, and K nutrients in Faxon fir across the three study sites.

Foliar	Mean	se	CV	N	Root	Mean	se	CV	N
Foliar C (mg g^-1^)	510.7	5.3	8.8%	72	Root C (mg g^-1^)	504.1	5.4	9.1%	72
Foliar N (mg g^-1^)	17.4	0.8	40.0%	72	Root N (mg g^-1^)	10.8	0.2	19.0%	72
Foliar P (mg g^-1^)	2.2	0.1	54.8%	72	Root P (mg g^-1^)	0.7	0.01	17.4%	72
Foliar K (mg g^-1^)	10.8	0.3	25.8%	72	Root K (mg g^-1^)	1.1	0.08	58.2%	72
Foliar C:N	33.0	1.2	31.9%	72	Root C:N	48.3	1.2	21.4%	72
Foliar C:P	283.6	13.8	41.2%	72	Root C:P	727.7	20.7	24.2%	72
Foliar N:P	8.5	0.2	30.7%	72	Root N:P	15.4	0.4	98.6%	72
Foliar N:K	1.7	0.06	22.0%	72	Root N:K	14.0	1.6	25.0%	72
Foliar P:K	0.2	0.009	38.9%	72	Root P:K	0.9	0.09	81.5%	72

Soil pH was measured using a mixture of air-dried soil sample and deionized water at a 1:2.5 (w:v) ratio with a pH meter (HI-9125, Hanna Instruments Inc., Woonsocket, RI). Soil water content (SWC, %) was calculated by drying fresh soil samples at 75°C to a constant weight for at least 48 h. Soil total N (TN, mg g^-1^) and organic C (SOC) were analyzed in air-dried samples previously passed through a 2 mm mesh sieve. TN was analyzed using the Kjeldahl digestion procedure (Gallaher et al., 1976). In turn, SOC was measured using the K_2_Cr_2_O_7_-H_2_SO_4_ calefaction method (Nelson and Sommers, 1983), while soil total potassium (TK, mg g^-1^) and phosphorus (TP, mg g^-1^) were measured using an ICP (ICAP6300). Additionally, soil C:N, C:P, N: P, N:K, and P:K ratios were calculated from the SOC: TN, SOC: TP, TN: TP, TN: TK, and TP: TK ratios (**Table 2**), respectively. Soil available P (AP, mg kg^-1^) and N (AN, mg kg^-1^), including NH_4_^+^-N and NO_3_^–^N concentrations, were measured in 10-g fresh soil samples by extraction with 2M KCl (Maynard et al., 1993), and using a continuous flow analyzer (SEAL AA3, Norderstedt, Germany).

**Table 2:** Descriptive statistics of soil C, N, P, K concentrations and stoichiometry across the study sites.

Sites	Wolong Natural Reserve	Miyaluo Natural Reserve	Wanglang Natural Reserve	*CV*
TN (mg g^-1^)	3.2 ± 0.1b	2.8 ± 0.3b	5.5 ± 0.4a	41.0%
TP (mg g^-1^)	0.80 ± 0.04b	0.76 ± 0.03b	1.1 ± 0.05a	30.0%
TK (mg g^-1^)	17.2 ± 0.6a	17.3 ± 0.9a	11.7 ± 0.6b	25.1%
AN (mg kg^-1^)	40.8 ± 2.4a	40.4 ± 7.3a	41.1 ± 3.8a	46.1%
AP (mg kg^-1^)	5.2 ± 0.6b	16.8 ± 3.3a	4.8 ± 0.5b	109.3%
Soil C: N	32.1 ± 1.3a	36.2 ± 1.2a	34.1 ± 1.3a	21.6%
Soil N: P	4.2 ± 0.2b	3.6 ± 0.3b	5.1 ± 0.4a	33.3%
Soil C: P	141.1 ± 7.5b	129.9 ± 9.7b	172 ± 16.2a	35.6%
Soil N: K	0.19 ± 0.01b	0.18 ± 0.03b	0.5 ± 0.06a	71.8%
Soil P: K	0.05 ± 0.002b	0.05 ± 0.004b	0.1 ± 0.01a	45.8%

TN: soil total N concentration, TP: soil total P concentration, TK: soil total K concentration, AN: soil available inorganic N, AP: soil available P. Values designated by the different letters are significantly different at *P* < 0.05 across the three study sites. N=72. mean ± se.

### Classification and measurement of ECM root traits

To explore the relationships between ECM traits and plant C:N, P:K nutrient stoichiometry, we built a new dataset (**Supplementary Table 1**) involving the formation and morphological differentiation of ECM fungi on root tips, including the colonization ratio of different hyphal exploration types, foraging length and area radiated by ECM root tips and extraradical mycelia, and ECM morphological diversity.

Root samples stored in 5% glycerin were gently washed under running tap water, and the soil particles adhering to the root tips were removed using fine forceps under a stereoscopic microscope. When the roots were covered with fungal mantles, they were classified as ectomycorrhiza, ECM, root tips; thus, at least 300 ECM root tips were identified in each soil block. The morphology of the ECM was determined using a photographic stereomicroscope (Leica, M205FA, Germany), and the macroscopic and microscopic characteristics of the mycorrhizae (i.e., ECM system, color, mantle surface structure, cystidia, emanating hyphae, and rhizomorphs), were identified based on Agerer (2006). We used the classification of Agerer (2001) and the Information System for Characterization and Determination of Ectomycorrhizae (DEEMY) database (http://www.deemy.de/) to assess ECM soil exploration types. Subsequently, ECM exploration types associated with Faxon fir were categorized into contact exploration with no emanates, short-distance exploration with short hyphae, and long-distance exploration with rhizomorphs, by morphological type identification by stereo microscopy, and root tips of each type at every elevation were counted. In all, 89,538 ECM root tips were recorded in the three study areas. The colonization ratio of the three exploration types was calculated relating the number of root tips of each type to the total number of root tips in each soil block. The morphological diversity index of the ECM root tips (MDI) was measured using Simpson’s index of diversity, as described by Lande (1996) and Matsuda and Hijii (2004).

**Figure 1:**
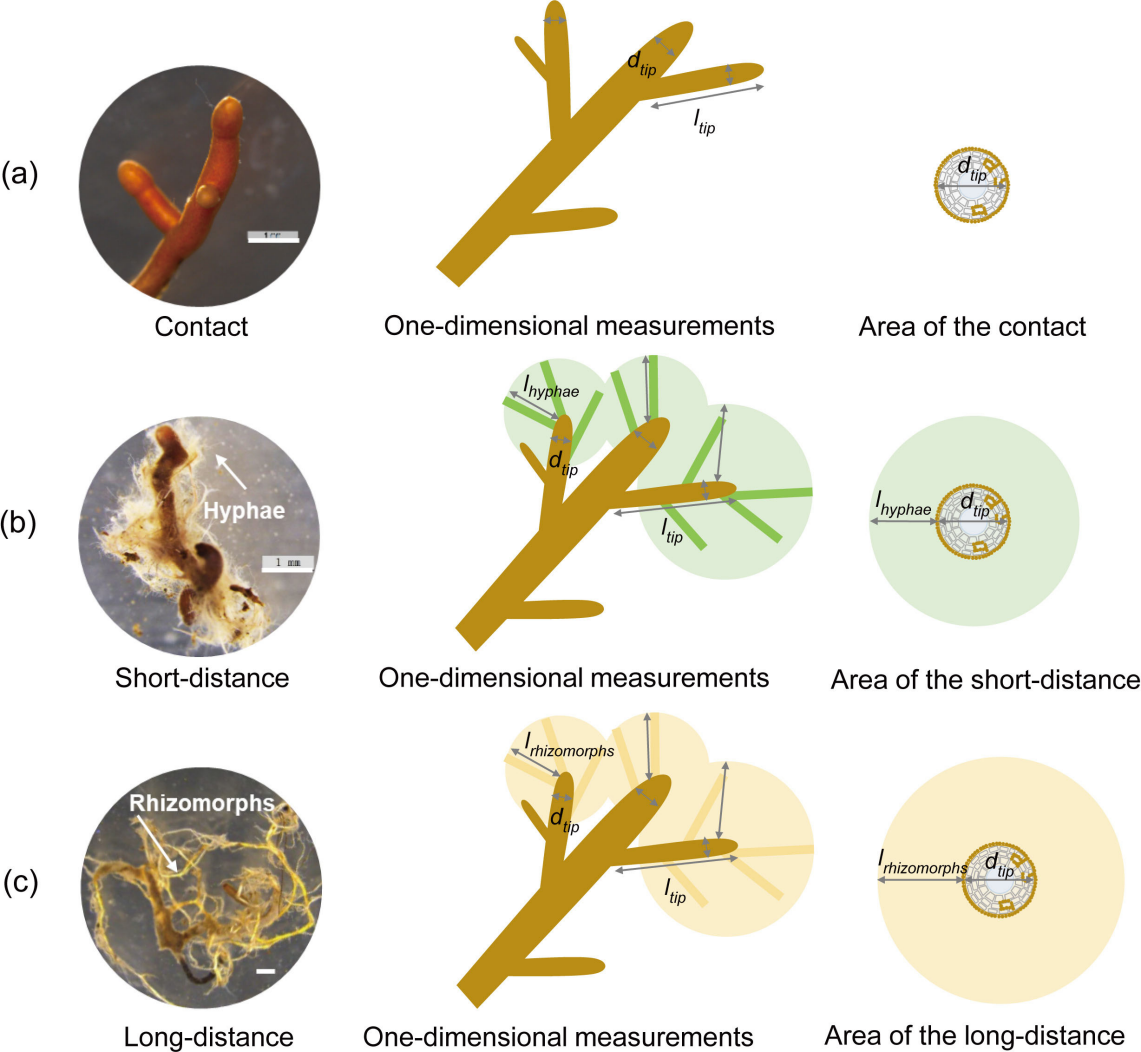
Quantification method of ectomycorrhizal foraging traits for different soil exploration types. **(a)** Contact exploration type; **(b)** Short-distance exploration type; **(c)** Long-distance exploration type. We took the area of the circle radiating from the radius of the extradical mycelium as the hyphal foraging area around the root tip. *d*_tip_, diameter of the distal ectomycorrhizal root tip; *l*_tip_, length of the distal ectomycorrhizal root tip; *l*_hyphae_, the maximum hyphal length on the root tip of short-distance exploration type; *l*_rhizomorph_, the maximum hyphal length on the root tip of long-distance exploration type. White horizontal bar: scale in 1 mm.


For representative ECM root samples of each morphotype in each soil block, three root tips were used for diameter (*d*, mm) and length (*l*, mm) measurements of ECM unramified ends and maximum length (*L*, mm) measurements of unramified ends emanating from hyphae/rhizomorphs using a photographic stereomicroscope (**Figure 1**). The diameter (RD) and length (RL) of the ECM root tips are the average values of all ECM tips in each soil block. In addition, the average maximum length of the ECM emanating from the distal root was measured in five replicates for each morphotype at the same elevation. We estimated the length and area radiated by the weight of the ECM emanating and root tip length (**Figure 1**), and the related ECM root tip number in each soil block (10×10×10 cm^3^), as follows:

$$Length\;(cm\;m^{-3})=\sum_{i=1}^{N}(L_{i}\times n_{i})\times 100$$

$$Area\;(\;m^{2}\;m^{-3})=\sum_{i=1}^{N}(\pi L_{i}^{2}\times n_{i})\times 0.001$$

where, *L_i_* represents the maximum length of the hyphae or rhizomorphs of ECM morphotype *i*; *n_i_* represents the number of ECM root tips of morphotype *i*, and *N* represents the total number of ECM morphology types in each soil block (10 cm ×10 cm ×10 cm).

The surface area of the ECM root tips (SA) was measured for samples in each soil block with the root tips determined as a combination of cylinders and hemispheres, as follows:


$$SA\;(m^{2}m^{-3})=\sum_{i=1}^{N}[(2\text{π}{\left(\frac{d_{i}}{2}\right)}^{2}+\text{π}\times d_{i}(l_{i}-d_{i}))\times n_{i}]\times 0.001$$

where *d_i_* represents the average diameter of the ECM root tips of morphotype *i*; *l_i_* represents the average length of the ECM root tips of morphotype *i*; *n_i_* represents the number of ECM root tips of morphotype *i*, and *N* represents the total number of ECM morphological types.

### Statistical analysis

All leaf and root chemical-trait data were tested for normality of distribution using the Lilliefors and Shapiro–Wilk tests, and the homogeneity of variance was tested using F and Levene’s tests in SPSS. 20.0. We performed a One-way ANOVA on these traits between the roots and foliage at the different study sites to examine the variation in C, N, P, and K concentrations and the stoichiometry in different tree tissues along the selected environmental gradient.

We used linear regression analysis to examine how plant C, N, P, and K concentrations and stoichiometry varied with elevation, as well as the relationships between these indices in roots and leaves. We explored how plant C, N, P, and K concentrations and stoichiometry varied with environmental resources at each site by performing a standardized major axis regression (SMA) to test the changing rate of plant element stoichiometry with soil element stoichiometry.

To assess the relative importance of the three groups (soil, climate, and ECM associations) for plant nutrient stoichiometry, we used a full model incorporating soil properties (C:N, C:P, N:P, N:K, P:K, AN, AP, pH, SWC), climate factors (MAT, MAP), and ECM functional traits (colonization ratio of hyphal exploration types, extraradical mycelium length and area, SA, RD, RL, MDI). The best predictors were selected, and the relative effect of each variable was calculated using the R package *MuMIn*. All explanatory and response variables were standardized using the *Z*-score before analysis, similar to the variance decomposition analysis (Gross et al., 2017). Model averaging was performed based on AICc weights when multiple models were selected. Additionally, model residuals were inspected for constant variance and normality.

To estimate the importance and explanation of ECM functional traits on tree tissue C:N:P:K stoichiometry, we used RandomForest analysis by defining two parameters: the number of trees in the forest (ntree) and the number of variables used per tree (mtry) (Delgado-Baquerizo et al., 2016). The standard for ntree defined in the package is 500, the standard value for mtry is one third of the total number of predictor variables. In RandomForest models, ECM functional traits served as predictors for tree tissue C, N, P, and K concentrations and stoichiometry. We also calculate the Spearman correlations between each two variables to estimate the relationships between ECM traits with plant C, N, P, K nutrients and stoichiometric ratios. We used R (4.3.1) for the statistical analyses and the generation of the figures.

## Results

### Leaf C, N, P, and K concentrations and stoichiometric ratios along the environmental gradient

**Figure 2:**
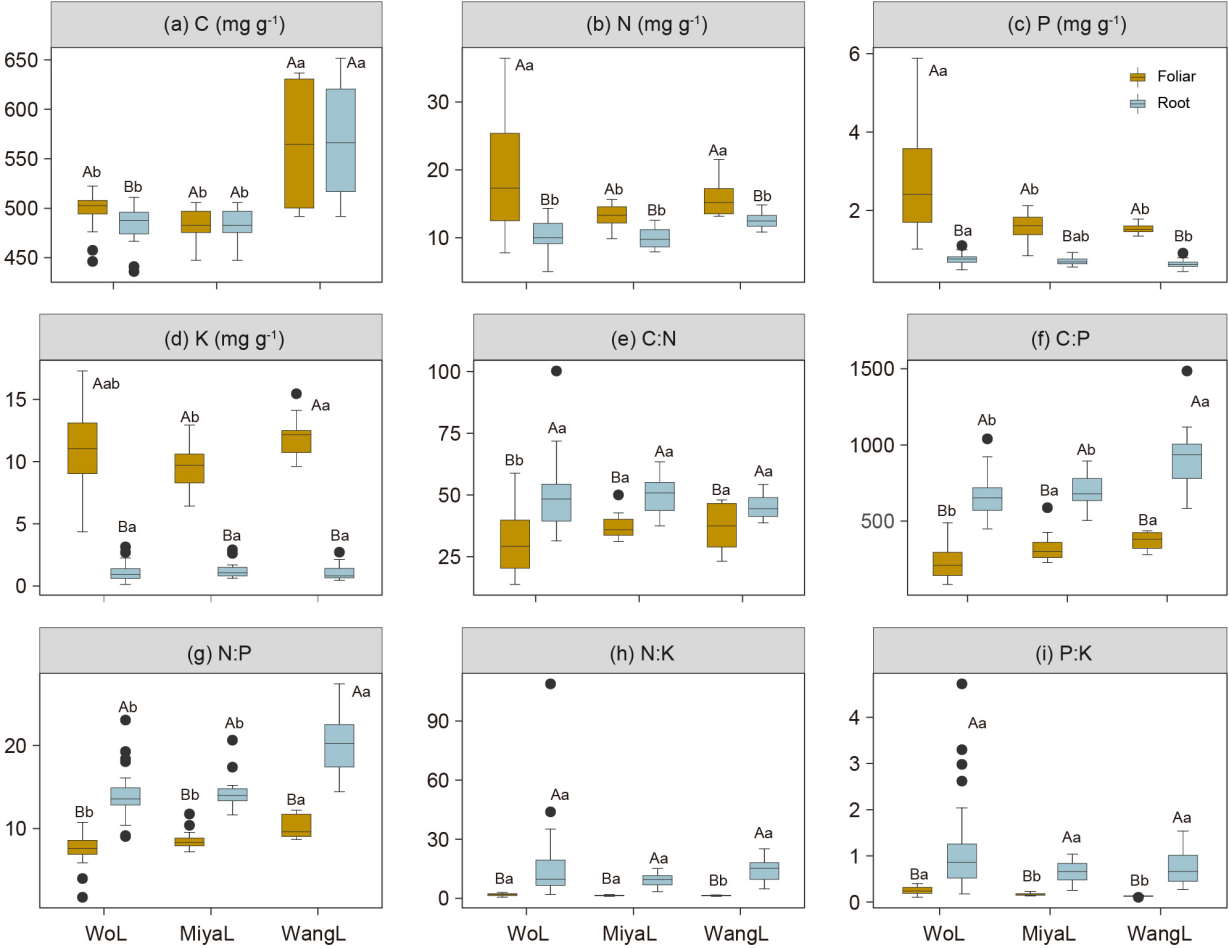
Foliar and root C, N, P and K concentrations and stoichiometry in Faxon fir across different sites. **(a)** C concentration; **(b)** N concentration; **(c)** P concentration; **(d)** K concentration; **(e)** C:N ratio; **(f)** C:P ratio; **(g)** N:P ratio; **(h)** N:K ratio; **(i)** P:K ratio; Different uppercase letters indicate significant differences between foliage and root at the 0.05 level (*P* < 0.05), and lowercase letters indicate significant differences between different sites at the 0.05 level (*P* < 0.05). Mean value ± standard error. Duncan’s multiple comparison test. WoL, Wolong Natural Reserve; MiyaL, Miyaluo Natural Reserve; WangL, Wanglang Natural Reserve.

**Figure 3:**
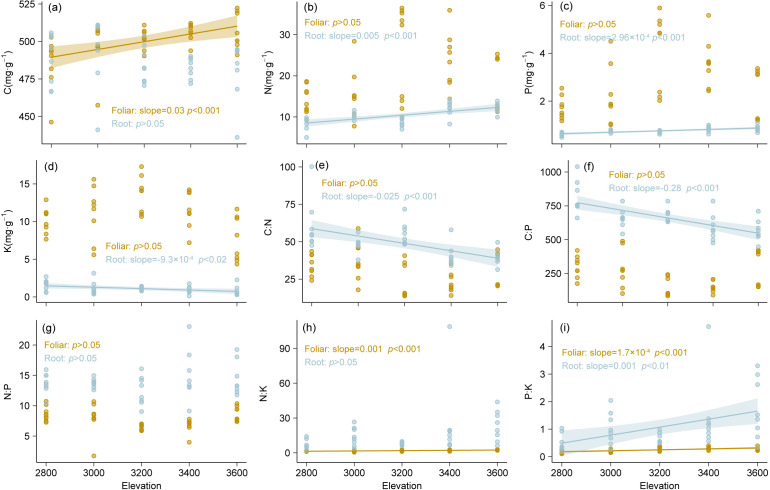
Patterns of foliar and root C, N, P and K concentrations and stoichiometry in Faxon fir along elevations. **(a)** C concentration; **(b)** N concentration; **(c)** P concentration; **(d)** K concentration; **(e)** C:N ratio; **(f)** C:P ratio; **(g)** N:P ratio; **(h)** N:K ratio; **(i)** P:K ratio.

Leaf N, P, and K concentrations were generally higher than those in the roots across the three study sites (**Figure 2**). Further, N and P were the two most variable leaf nutrient concentrations, with CV values of 40% and 54.8%, respectively, whereas K concentration, and N:P and P:K ratios were the most variable parameters in the roots, with CV of 58.2%, 98.6%, and 81.5%, respectively (**Table 1**). Additionally, we detected significant differences in the leaf C:N, N:K, and P:K ratios and in the leaf and root C:P and N:P ratios among latitudinal sites, whereas root C:N, N:K, and P:K ratios were not significantly different (**Figures 2e-i**). Leaf C concentration significantly increased with increasing elevation, whereas root N, P, and K concentrations decreased (**Figures 3a-d**). Similarly, root C:N and N:P ratios decreased with increasing elevation (**Figures 3e, f**), whereas the leaf N:K, P:K, and root P:K ratios increased with increasing elevation (**Figures 3h, i**).

### Relationships of C, N, P, K concentrations and stoichiometry among root, foliage, and soil

**Figure 4:**
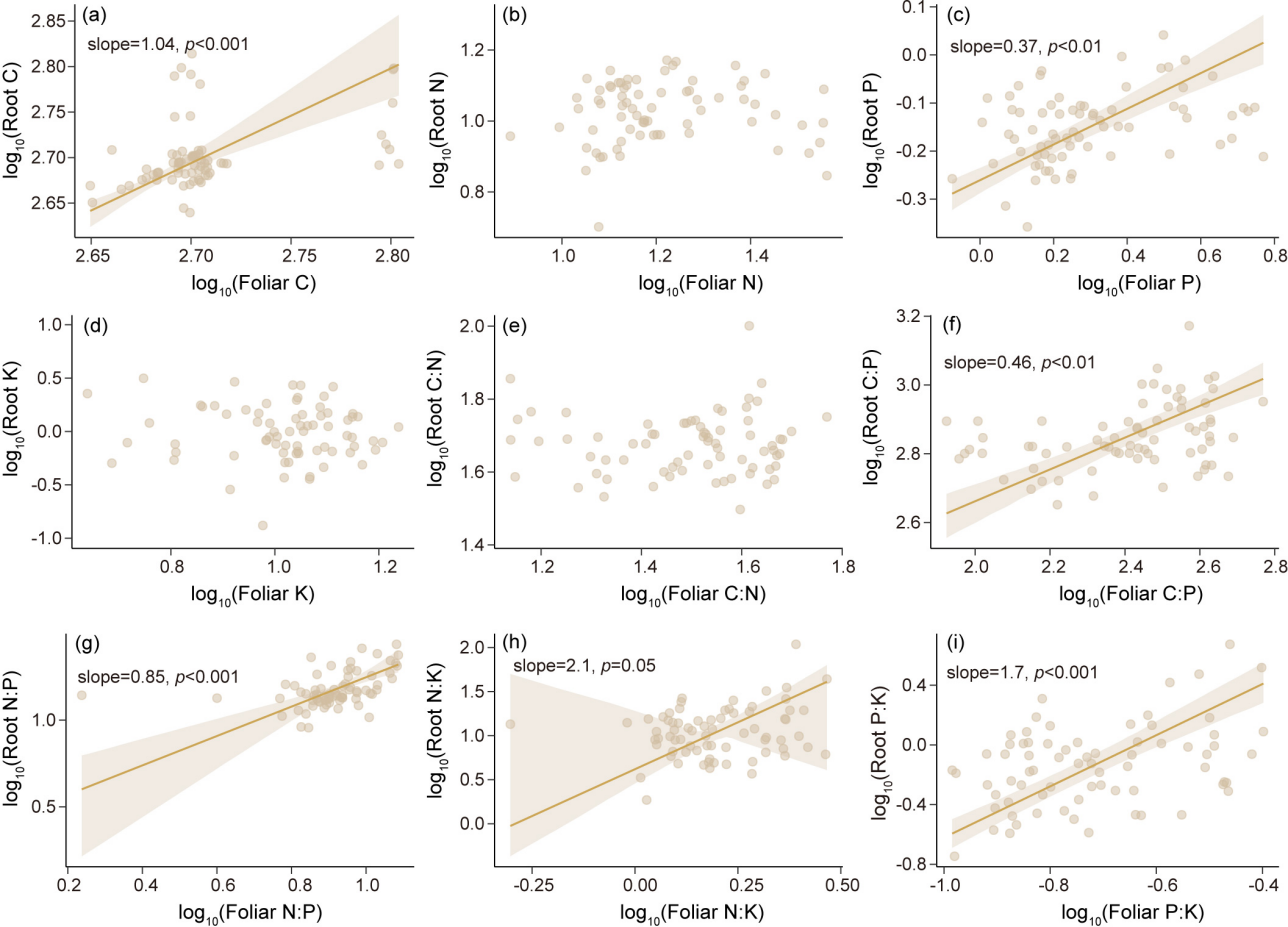
Relationships of foliar and root C, N, P, and K concentrations and stoichiometry by linear regression. Data of the x- and y-axis were log-transformed. **(a)** C concentration, **(b)** N concentration, **(c)** P concentration, **(d)** K concentration, **(e)** C:N ratio, **(f)** C:P ratio,**(g)** N:P ratio,**(h)** N:K ratio,**(i)** P:K ratio.

**Figure 5:**
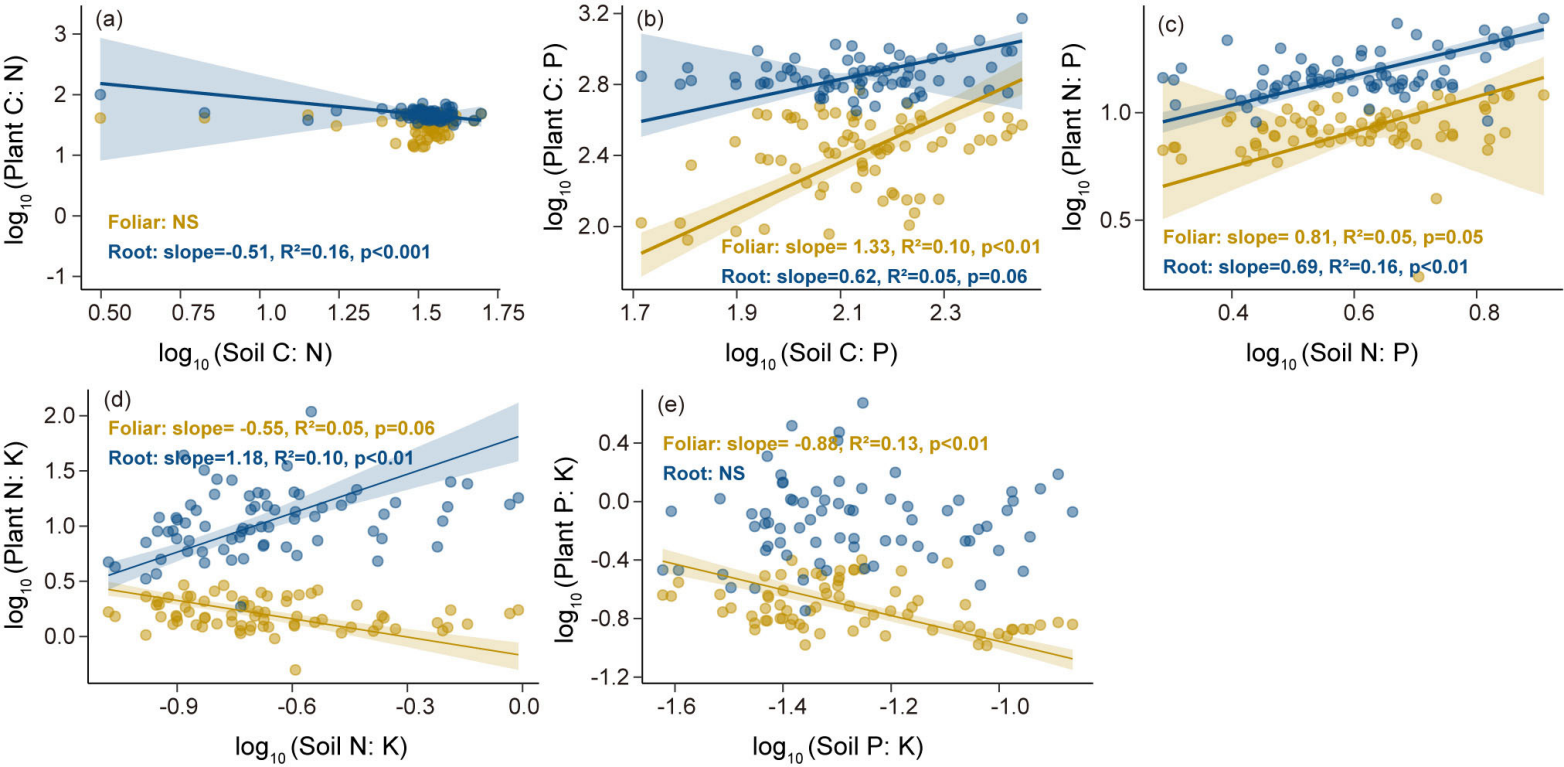
Standardized Major Axis (SMA) regression analysis of foliar and root C:N:P:K stoichiometry in relation to soil C:N:P:K stoichiometry. **(a)** C:N ratio, **(b)** C:P ratio, **(c)** N:P ratio, **(d)** N:K ratio, **(e)** P:K ratio. Data of the x- and y-axis were log10-transformed.

Leaf C and P concentrations (**Figures 4a, c**) and C:P, N:P, N:K, and P:K ratios (**Figures 4f-i**) significantly and positively correlated with those in the roots. According to the SMA results, there were significant negative correlations between root and soil C:N, between root and soil N:P, and between leaf and soil P:K (**Figures 5a, c, e**); conversely, leaf and root N:P ratios positively correlated with soil N:P ratio, and the slope of the former (slope = 1.33, p <0.01) was higher than that of the latter (slope = 0.61, p = 0.06). Meanwhile, leaf and root N:P ratios positively correlated with soil N:P ratio, with slopes of 0.81 (p = 0.05) and 0.69 (p <0.01) (**Figure 5c**), respectively. In turn, the root N:K ratio positively correlated with the soil N:K ratio (slope = 1.18, p <0.01), whereas the leaf N:K ratio negatively correlated with the soil N:K ratio (slope =-0.55, p = 0.06) (**Figure 5d**).

### Effects of climate, soil, and ECM variables on C, N, P, K contents and stoichiometry in leaves and roots of Faxon fir trees

**Figure 6:**
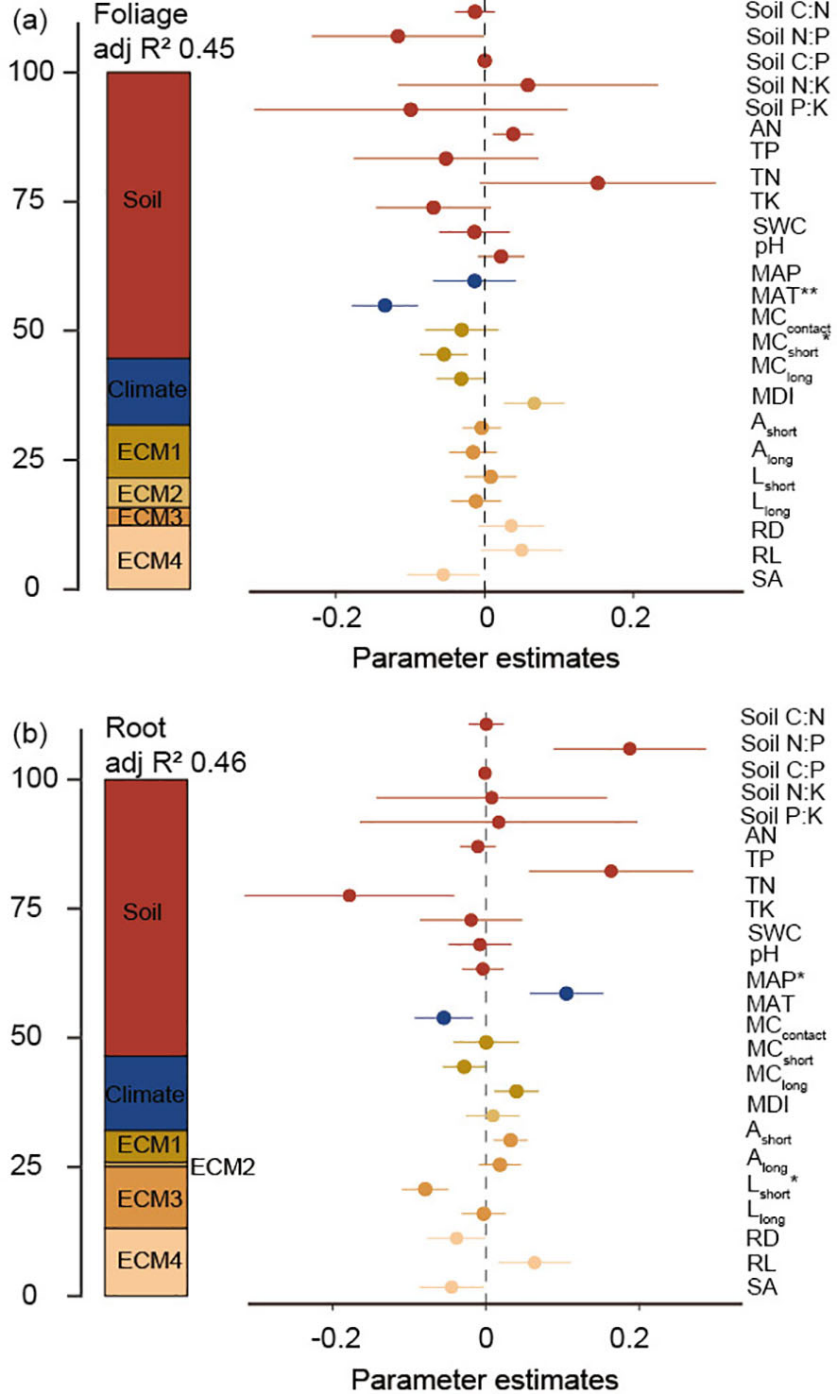
Effect of soil variable, climate, and ECM functional traits on foliage **(a)** and root **(b)** stoichiometry.. Average parameter estimates (standardized regression coefficients) of model predictors, associated 95% confidence intervals, and relative importance of each factor, expressed as the percentage of explained variance. The adjusted (adj.) R^2^ of the averaged model and the P value of each predictor are given as: *P < 0.05; **P < 0.01. ECM1: represents the formation of ECM fungi on root tips, including MC_contact_, MC_short_, and MC_long_; ECM2: represents ECM morphology diversity, i.e. MDI; ECM3: foraging-space mediated by ECM mycelium, including A_short_, A_long_, L_short_, and L_long_; ECM4: foraging traits of ECM root tips, including RD, RL, and SA.

Soil variables were the most important factors in explaining the variation in root and leaf nutrient stoichiometry, followed by ECM functional traits (**Figure 6**). Indicators of growth-related (colonization ratio) and morphological traits (RD, RL, and SA) of ECM root tips were the most important factors explaining the variation in leaf nutrient stoichiometry, whereas the morphological traits of ECM root tips and extraradical mycelia were the most important factors explaining root nutrient stoichiometry (**Figure 6**). As for climatic factors, MAT and MAP largely explained the variation in leaf (p <0.01) and root (p <0.05) nutrient stoichiometry (**Figure 6**).

**Figure 7:**
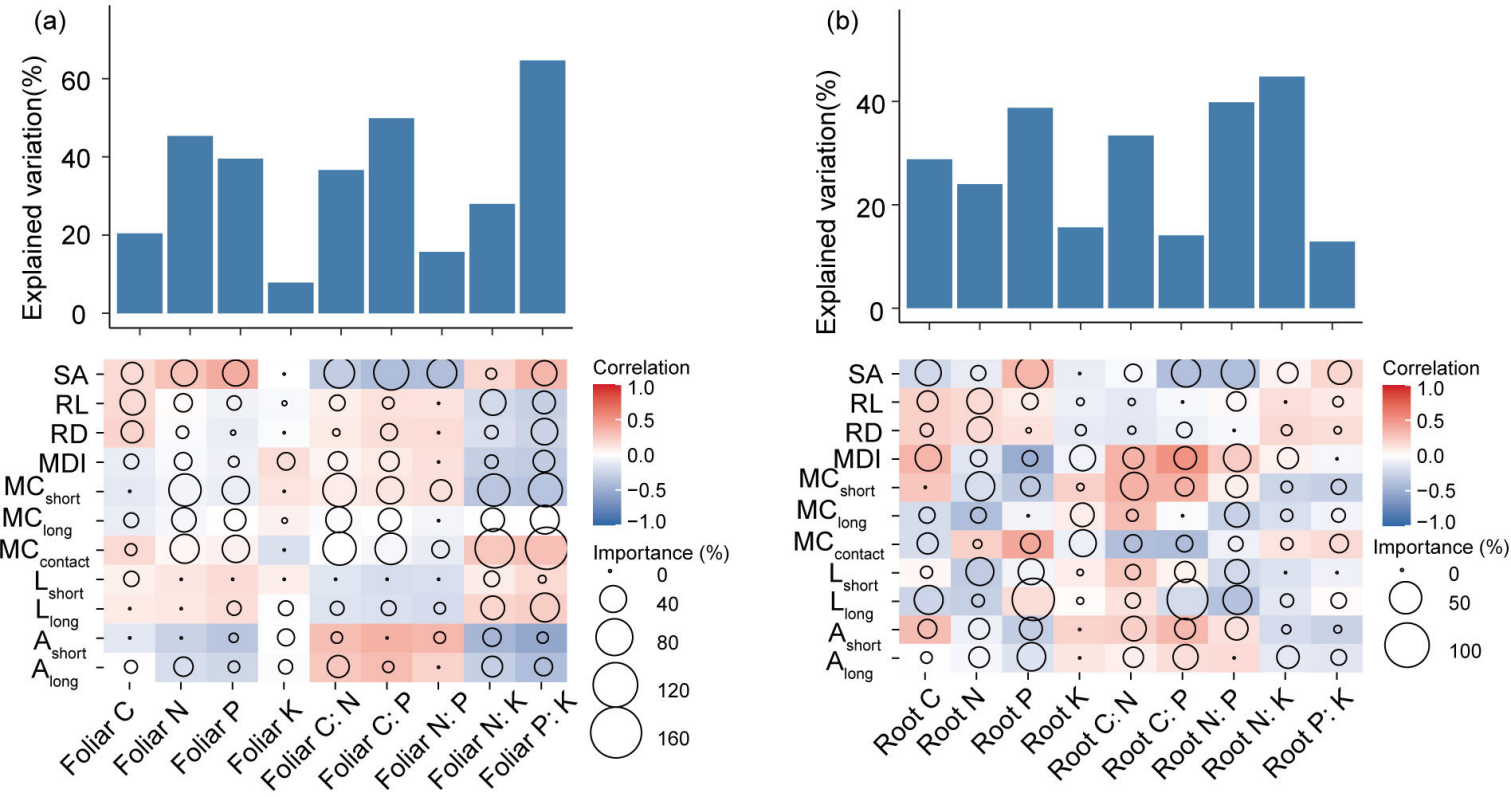
Contributions of ECM properties to the dissimilarities of foliar **(a)** and root **(b)** C, N, P, K contents and stoichiometry based on correlation and best multiple regression model. We examined the correlation of the values of C, N, P, and K concentrations and stoichiometry with the differences in ECM properties and identified the major predictors. Circle size represents the variable importance (that is, the proportion of explained variability calculated via multiple regression modeling and variance decomposition analysis). Colors represent Spearman correlations. MC_contact_, colonization ratio of contact exploration type; MC_short_, colonization ratio of short-distance exploration type; MC_long_, colonization ratio of long-distance exploration type; A_short_, area radiated by the short-distance exploration type; A_long_, area radiated by the long-distance exploration type; MDI, ECM morphology diversity index; L_short_, hyphae length density of the short-distance exploration type; L_long_, length density of the long-distance exploration type; RD, diameter of ECM root tips; RL, length of ECM root tips; SA, superficial area of ECM root tips.

According to the results of Random Forest analysis, ECM traits were closely correlated with plant C:N, and P stoichiometry, but their relationships with tree K concentrations were weak (**Figure 7**). Leaf C strongly and positively correlated with the morphological traits of ECM root tips (RD, RL, and SA), whereas root C was most closely related to morphological diversity (MDI) and L_long_. ECM growth-related traits (MC_contact_, MC_short_, and MC_long_) were the most important factors explaining leaf N and P concentrations, and C:N, C:P, N:K, and P:K ratios. ECM foraging range-related traits (e.g., length and surface area radiated by hyphae) were more closely related to root C, N, and P concentrations and their elemental ratios. Lastly, root K was most affected by MDI, MC_contact_, and MC_long_.

## Discussion

### Patterns of foliage and root C, N, P, and K nutrients along the selected environmental gradient

*Faxon fir is significantly K-limited.* Faxon fir leaves had a higher C concentration (510.7 ± 5.3 mg g^-1^; **Table 1**) than the global average (464 mg g^-1^; Elser et al., 2000), which may enhance stress adaptation in subalpine environments (Körner, 2016). Leaf and root N and P concentrations of Faxon fir were comparable to those of global plants (Han et al., 2005; Zhao et al., 2020); however, K concentrations (leaves: 10.8 ± 0.3 mg g^-1^, roots:1.1 ± 0.08 mg g^-1^, **Table 1**) were much lower than other plants from natural communities in China, for example, leaf K concentrations in 2781 species of higher plants from natural communities in China averaged 18.3 mg g^-1^ (Li et al., 2021), root K concentration is approximately 5.1 mg/g for 551 plant species in eight forest ecosystems in China (Zhao et al., 2020). The high N:K ratio (>2.1) indicates K limitation (Olde Venterink et al., 2003), suggesting K scarcity restricts Faxon fir growth in subalpine ecosystems (**Table 1**).

*More N, P, and K nutrients are allocated to leaves than to roots in Faxon fir.* Faxon fir allocated more N, P, and K to leaves than roots (**Figure 2**; **Supplementary Figure S1**), with higher P accumulation rates in leaves than in roots (**Figure 4**). Such allocation patterns align with the optimal partitioning theory (Liu et al., 2021; McCarthy and Enquist, 2007; Reich and Oleksyn, 2004), wherein tree species optimize nutrient retention in photosynthetic tissues to enhance nutrient conservation. This strategy may mitigate potential nutrient limitations in cold subalpine forests, where low microbial activity and reduced root foraging efficiency constrain soil nutrient availability (Domisch et al., 2002; Graça and Poquet, 2014). As an evergreen dark conifer species, Faxon fir may require more nutrients to maintain the long-term physiological activity of leaves (Zhang et al., 2018), consistent with the temperature-plant physiology hypothesis (Reich and Oleksyn, 2004). This foliar prioritization may compensate for low photosynthetic and metabolic rates of Faxon fir in cold climates (Craine, 2005; Kerkhoff et al., 2006; Palmroth et al., 2013; Wright et al., 2003), following the functional compensation theory. However, contrasting evidence suggests trees may allocate more nutrients to roots for efficient soil foraging (Zhang et al., 2018), highlighting Faxon fir’s distinct foliar-centric strategy. Notably, the coordinated C:P, N:P, and N:K ratios between roots and leaves (**Figure 4**) imply internal nutrient homeostasis in Faxon fir (Chen et al., 2023; Craine, 2005; Sardans et al., 2021; Wang et al., 2019), potentially driving root systems to intensify nutrient uptake activity to sustain foliar demand. Across elevations, root N, P, and K exhibited greater variability than foliar nutrients (**Figure 3**), underscoring aboveground nutrient stability and belowground sensitivity to environmental change. Root N and P increased with elevation (**Figures 3b, c**), suggesting a cold-adaptation strategy via enhanced nutrient retention (Chapin et al., 1986; Weih and Karlsson, 2001), supported by declining root C:N and C:P ratios (**Figure 3**). This aligns with the temperature-plant physiology hypothesis, wherein plants elevate N and P to offset low-temperature metabolic constraints (Gao et al., 2023; Reich and Oleksyn, 2004). Conversely, declining root K at higher elevations (**Figure 3**) may reflect metabolic downregulation, potentially impairing cold resistance. Collectively, Faxon fir employs a dual elevational strategy: foliar dominance for nutrient conservation and root adjustments for cold adaptation. This stoichiometric flexibility likely represents an adaptive trade-off to sustain viability in harsh environments, though it may constrain long-term resilience under climate change.

### Effects of environmental factors on leaf and root C, N, P, and K balance

*The stoichiometric plasticity of Faxon fir in changing environments*. Our results indicate that plant nutrient status in cold coniferous forests was more strongly influenced by soil fertility than by climatic factors (**Figure 6**). Specifically, leaf (C:P, N:P, N:K, P:K) and root (C:N, C:P, N:P, N:K) stoichiometric ratios varied significantly with soil nutrient levels (**Figure 5**), demonstrating weak stoichiometric homeostasis in Faxon fir—contrary to the predictions of Stoichiometric Homeostasis Theory (Sardans et al., 2015; Sterner and Elser, 2017; Zhang et al., 2013). This suggests that Faxon fir growth is highly sensitive to soil conditions and exhibits stoichiometric plasticity in nutrient-limited environments, deviating from the conservative strategies observed in other tree species (Bowman et al., 1993; Müller et al., 2017; Zheng et al., 2021). Such plasticity likely reflects adaptive mechanisms to optimize nutrient uptake and utilization under fluctuating conditions (Zhang et al., 2020). Climate change further disrupts nutrient balance, with MAT driving foliar stoichiometric variation and MAP influencing root stoichiometry (**Figure 6**). Warming reduced N and P concentrations and N:K and P:K ratios in both tissues while increasing C:N ratios (**Supplementary Figure S2**), consistent with enhanced physiological activity under higher temperatures (Tian et al., 2018; Wang et al., 2015). Furthermore, warming can facilitate the utilization of N and P by stimulating microbial and root-associated fungal activity (e.g., mycorrhizal symbionts), thereby modifying nutrient acquisition dynamics (Kwatcho Kengdo et al., 2022; Zhou et al., 2016). Given the positive correlations between foliar N and P concentrations, N:K and P:K ratios, and MAP (**Supplementary Figure S2**), projected warming and drying could further constrain nutrient availability for Faxon fir. Collectively, Faxon fir appears to adapt by dynamically balancing tissue-specific C:N:P:K stoichiometry, though its long-term resilience under climate change remains uncertain.

### Importance of ectomycorrhizal function traits on leaf and root C, N, P, and K balance

The phenotypic diversity of ECM fungi represents a critical biological indicator reflecting host plant adaptation to environmental resource heterogeneity (van der Linde et al., 2018; Jörgensen et al., 2023). Our systematic analysis of ECM morphological functional traits demonstrates robust correlations with nutrient flux variations in Faxon fir, exhibiting particularly strong associations with root C:N:P:K stoichiometric balance across environmental gradients (**Figures 6**, **7**). According to the C-nutrient trade between host tree and ECM fungi, it is certain that there are close associations between ECM functional traits and N, P, and K contents in plant tissues (Köhler et al., 2018; Smith and Read, 2010; van der Linde et al., 2018; Zhu et al., 2023); however, there is little evidence to support this statement. Importantly, Our results demonstrate the importance of ECM morphological traits in regulating the C, N, P, and K stoichiometric balance in Faxon fir.

*ECM one- and two-dimensional foraging traits could predict the stoichiometric dynamics in Faxon fir.* In this study, we found that foliar nutrient stoichiometry was correlated with the formation and morphological differentiation of ECM root tips, whereas that of roots was related to the nutrient-foraging space radiated by the ECM mycelium (**Figure 6**). This is because the relationships between leaf- and root-ECM systems mainly involve C-nutrient exchange, and C transport from the leaves determines the colonization and morphological differentiation of ECM fungi on root tips (Hobbie, 2006). Our findings suggest that the growth of ECM species may serve as a predictive indicator for aboveground C-nutrient allocation in host plants, while reciprocally, fluctuations in host stoichiometric ratios (C:N:P:K) potentially regulate ECM proliferation, though the absence of ECM species identification precludes mechanistic interpretation at the taxonomic level. The nutrient status of roots is closer to morphological diversity of ECM extraradical mycelium (**Figure 7b**), as such diversity directly reflects the functional heterogeneity in ECM nutrient foraging strategies (Agerer, 2001). Specifically, ECM taxa specialized in short-distance exploration can secrete N degradation-related enzymes (Tedersoo et al., 2012), such as proteases, which may be an important part of their ability to mediate N nutrient exchange between the root-ECM association. In contrast, ECM taxa with long rhizomorphs for long-distance exploration are dominated by large-scale nutrient space occupation (Weigt et al., 2012), capturing P through physical space expansion (Agerer, 2001; Smith and Read, 2010). Therefore, the differentiation of ECM short hyphae and long rhizomorph may contribute to the fluctuation of C:N, C:P ratios of tree species, respectively (**Figure 7b**). Currently, there is little direct evidence on the relationship between ECM fungi and host C:N:P:K stoichiometric ratios. Overall, our study illustrates the close associations between ECM functional traits and plant C:N:P:K stoichiometry; furthermore, it shows that ECM one- and two-dimensional foraging pathways can effectively predict and regulate the nutrient limitation status in tree species.

*Foraging length of ECM extraradical mycelium potentially regulate K nutrient in Faxon fir.* Ectomycorrhizal traits explained most of the variation in leaf P:K and root N:K ratios (**Figure 7**), suggesting that ECM functional attributes are associated with the transportation and regulation of water and solute in the host. It has been reported that ECM taxa with hydrophobic rhizomorphs thrive in low-K soils, whereas hydrophilic hyphae are used in nutrient acquisition in high-K soils (Agerer, 2001; Smith and Read, 2010; van der Linde et al., 2018). Under drought stress, ECM hyphae could enhance host water and K+ acquisition (Garcia et al., 2014), while potentially reducing N and P translocation to the host (Pena and Polle, 2014; Khullar and Reddy, 2019), thereby inducing fluctuations in host N:K and N:P stoichiometric ratios. Thus, ECM functional traits seemingly mediate K uptake and transportation along the soil-root-leaf continuum. Specifically, the foraging length of ECM extraradical mycelium caused a decline in foliar N:K and P:K ratios (**Figure 7**), indicating that ECM extraradical mycelium enhanced water and solute regulation in trees.

*Limitations and Future Directions*. Although this study established empirical linkages between ECM morphological traits and host stoichiometric traits, several limitations must be acknowledged. Most notably, the high plasticity of ECM species and their phenotypic diversity in response to environmental gradients (van der Linde et al., 2018; Jörgensen et al., 2023) complicates trait-function relationships in host-symbiont interactions. While our investigation focused solely on ECM phenotypic traits in relation to host C-nutrient dynamics—without accounting for species-specific ecological functions—the results clearly demonstrate strong associations between ECM fungal proliferation and foliar nutrient stoichiometry (**Figures 6**, **7**). Furthermore, the relationships between ECM fungal occurrence frequency (a growth indicator), nutrient foraging length and area (morphological differentiation indicators), and host nutrient dynamics and stoichiometry exhibited substantial variability (**Figures 6**, **7**). Given the persistent inconsistencies between molecular and morphological characterization methods, as well as the inherent limitations of each approach when used independently, we emphasize the necessity of integrating ECM species identification through molecular sequencing with phenotypic trait analysis to fully elucidate ECM-mediated nutrient regulation mechanisms in host trees.

## Conclusions

We found that K was the most limiting nutrient for the roots and foliage of Faxon fir. Generally, Faxon fir allocates more nutrients to the leaves, suggesting that aboveground growth is prioritized, which may result in a greater demand on root nutrient acquisition. Along environmental gradients, Faxon fir adopts a conservative strategy, whereby it retains nutrients at high elevations to maintain growth and metabolism. Root and foliar nutrient stoichiometry (e.g., C:N, C:P, N:P, N:K, and P:K ratios) varies significantly with those of the soil, resulting in high stoichiometric plasticity in Faxon fir. Our results allow us to predict the improvement of N and P utilization in Faxon fir under warming and drying conditions, and an imbalance of water and solute transportation mediated by K. Furthermore, we found that the formation and morphological differentiation of ECM fungi play an important role in the nutrient stoichiometric balance above and belowground. Functional traits of ECM root tips are intimately associated with foliar nutrient contents and stoichiometry, while those of roots can be predicted by the multidimensional foraging area of ECM mycelia.

## Data Availability

The datasets presented in this study can be found in online repositories. The names of the repository/repositories and accession number(s) can be found in the article/**Supplementary Material**.

## Supplementary material

Supplementary Figure 1 Ordination of Principal Component Analysis (PCA) showing the C, N, P, K concentrations and stoichiometry between foliage and root tissues. The results are expressed as a biplot, where the distance and direction from the axis center have the same meaning for C, N, P, K elements and stoichiometry. Numbers in parentheses represent variations explained by the first two principal components (PC).

Supplementary Figure 2 Correlation coefficient matrix of soil variables, climate factors, and C, N, P, K nutrients of root and leaf. AN, soil available inorganic N; AP, soil available P; TP, soil total P concentration; TN, soil total N concentration; TK, soil total K concentration; SWC, soil water content; MAP, mean annual precipitation; MAT, mean annual temperature *, *p* < 0.05, **, *p* < 0.01, ***, *p* < 0.001.

Supplementary Table 1 Descriptive statistics of ECM morphological traits in Faxon fir across the three study sites. MC_contact_, colonization ratio of contact exploration type; MC_short_, colonization ratio of short-distance exploration type; MC_long_, colonization ratio of long-distance exploration type; A_short_, area radiated by the short-distance exploration type; A_long_, area radiated by the long-distance exploration type; MDI, ECM morphology diversity index; L_short_, hyphae length density of the short-distance exploration type; L_long_, length density of the long-distance exploration type; RD, diameter of ECM root tips; RL, length of ECM root tips; SA, superficial area of ECM root tips.
