# Supplementary material for: Ectomycorrhizal morphology and soil nutrient status control the C: N: P: K stoichiometry in Faxon fir (Abies fargesii var. faxoniana), in the subalpine forests of Southwest China
Source: Front Plant Sci. 2025 May 16;16:1549476. doi: 10.3389/fpls.2025.1549476 (PMC12122739; doi:10.3389/fpls.2025.1549476)
Supplement: Supplementary Figure 1 — Ordination of Principal Component Analysis (PCA) showing the C, N, P, K concentrations and stoichiometry between foliage and root tissues. The results are expressed as a biplot, where the distance and direction from the axis center have the same meaning for C, N, P, K elements and stoichiometry. Numbers in parentheses represent variations explained by the first two principal components (PC). [file DataSheet1.pdf]

Supplementary materials

Table 1 Descriptive statistics of ECM morphological traits in Faxon fir across the three study sites.

ECM traits	Mean	se	CV (%)	N
$MC_{contact}$ (%)	30.4	0.02	64.7	72
MC_{short} (%)	12.1	0.01	89.1	72
MC_{long} (%)	18.5	0.02	75.96	72
MDI	0.88	0.009	9.59	72
A_{short} (m^2 m^{-3})	8.51	2.16	215.4	72
A_{long} (m^2 m^{-3})	92.1	15.91	146.6	72
L_{short} (cm m^{-3})	51.29	6.39	105.8	72
L_{long} (cm m^{-3})	215.54	27.97	110.1	72
RD (mm)	0.23	0.01	49.6	72
RL (mm)	2.18	0.15	59.8	72
SA (m^2 m^{-3})	2.36	0.21	76.2	72

$MC_{contact}$: colonization ratio of contact exploration type; MC_{short}: colonization ratio of short-distance exploration type; MC_{long}: colonization ratio of long-distance exploration type; A_{short}: area radiated by the short-distance exploration type; A_{long}: area radiated by the long-distance exploration type; MDI: ECM morphology diversity index; L_{short}: hyphae length density of the short-distance exploration type, L_{long}: length density of the long-distance exploration type, RD: diameter of ECM root tips, RL: length of ECM root tips; SA: superficial area of ECM root tips.

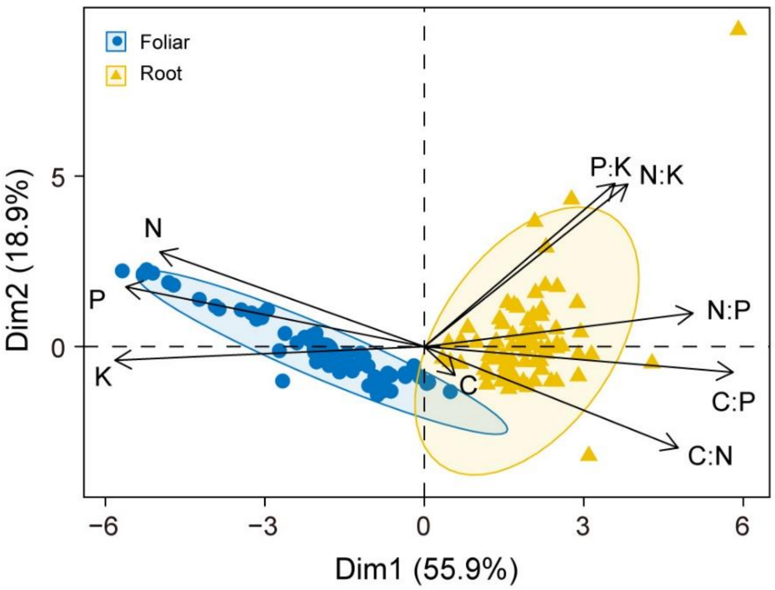

Fig.1 Ordination of Principal Component Analysis (PCA) showing the C, N, P, K concentrations and stoichiometry between foliage and root tissues. The results are expressed as a biplot, where the distance and direction from the axis center have the same meaning for C, N, P, K elements and stoichiometry. Numbers in parentheses represent variations explained by the first two principal components (PC).

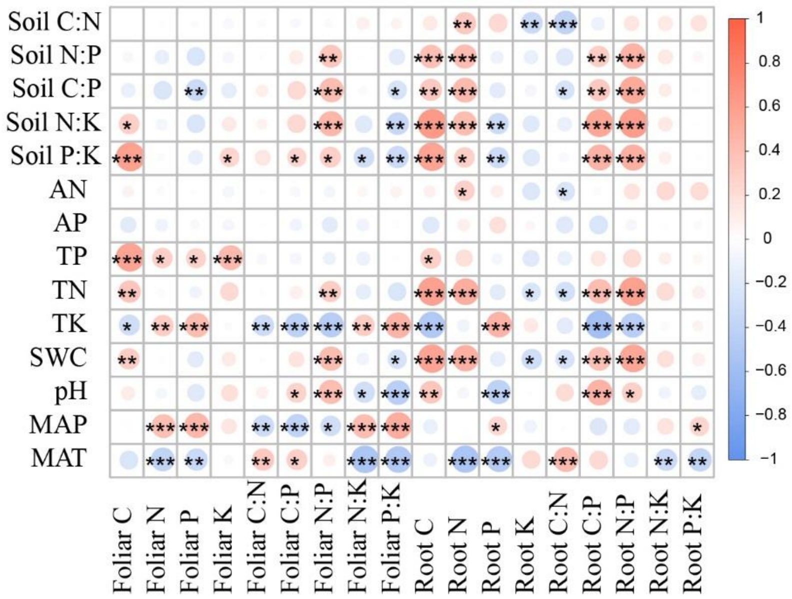

Fig.2 Correlation coefficient matrix of soil variables, climate factors, and C, N, P, K nutrients of root and leaf. AN: soil available inorganic N, AP: soil available P, TP: soil total P concentration, TN: soil total N concentration, TK: soil total K concentration, SWC: soil water content, MAP: mean annual precipitation, MAT: mean annual temperature *, $p < 0.05$, **, $p < 0.01$, ***, $p < 0.001$.
